# Conductive Hybrid Crystal Composed from Polyoxomolybdate and Deprotonatable Ionic-Liquid Surfactant

**DOI:** 10.3390/ijms17070994

**Published:** 2016-06-23

**Authors:** Jun Kobayashi, Ryosuke Kawahara, Sayaka Uchida, Shinichi Koguchi, Takeru Ito

**Affiliations:** 1 Department of Chemistry, School of Science, Tokai University, 4-1-1 Kitakaname, Hiratsuka 259-1292, Japan; j.koba92@gmail.com (J.K.); koguchi@tokai-u.jp (S.K.); 2 Department of Basic Science, Graduate School of Arts and Sciences, The University of Tokyo, 3-8-1 Komaba, Meguro-ku, Tokyo 153-8902, Japan; 9770832063@mail.ecc.u-tokyo.ac.jp (R.K.); csayaka@mail.ecc.u-tokyo.ac.jp (S.U.)

**Keywords:** inorganic-organic, hybrid crystal, polyoxometalate, ionic-liquid, surfactant

## Abstract

A polyoxomolybdate inorganic-organic hybrid crystal was synthesized with deprotonatable ionic-liquid surfactant. 1-dodecylimidazolium cation was employed for its synthesis. The hybrid crystal contained δ-type octamolybdate (Mo_8_) isomer, and possessed alternate stacking of Mo_8_ monolayers and interdigitated surfactant bilayers. The crystal structure was compared with polyoxomolybdate hybrid crystals comprising 1-dodecyl-3-methylimidazolium surfactant, which preferred β-type Mo_8_ isomer. The less bulky hydrophilic moiety of the 1-dodecylimidazolium interacted with the δ-Mo_8_ anion by N–H···O hydrogen bonds, which presumably induced the formation of the δ-Mo_8_ anion. Anhydrous conductivity of the hybrid crystal was estimated to be 5.5 × 10^−6^ S·cm^−1^ at 443 K by alternating current (AC) impedance spectroscopy.

## 1. Introduction

Ionic-liquids exhibit characteristic conductive or catalytic properties, and enable us to construct functional hybrid materials [1,2,3]. Ionic-liquid species often contain imidazolium moiety in their molecular structures. Inorganic-organic materials comprising imidazolium ionic-liquid have been explored as ionic or proton conductors [4,5]. As for inorganic components in hybrid conducting materials, polyoxometalate anions are effective candidates [6,7,8,9,10,11,12,13,14,15,16,17]. Polyoxometalates and ionic-liquids have been successfully hybridized [18,19,20,21,22], and some of them exhibit promising conductive properties [15,18].

In such polyoxometalate hybrids with ionic-liquids, the structure and arrangement of molecular components should be precisely controlled for the emergence of characteristic functions. To construct well-ordered polyoxometalate-ionic liquid hybrids, utilizing structure-directing species such as surfactant molecules [23,24,25] is advantageous. Polyoxometalate anions have been organized by surfactant cations to form inorganic-organic hybrids [26,27,28,29,30,31,32,33,34,35,36,37,38,39,40] and single crystals [41,42,43,44,45,46,47,48,49,50,51,52,53,54,55,56,57]. These polyoxometalate-surfactant hybrids allow flexible selection of the ionic components, which leads to precise engineering of the structure and function. In addition, polyoxometalate single crystals hybridized with ionic-liquid surfactants have also been reported [45,46,52,53,54].

**Figure 1:**
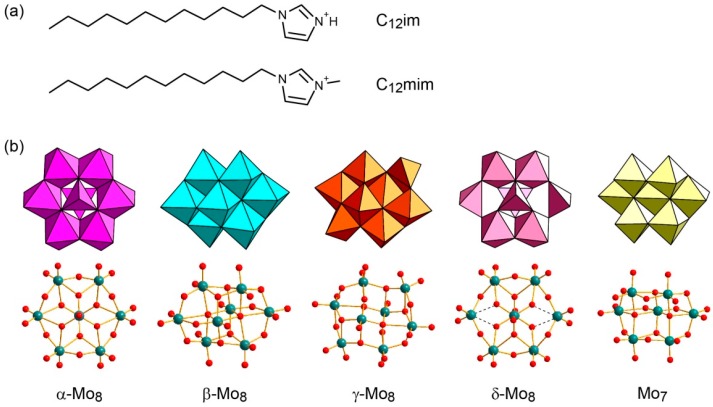
Molecular structures of ionic-liquid surfactants and polyoxomolybdates: (**a**) 1-dodecylimidazolium (C_12_im) and 1-dodecyl-3-methylimidazolium (C_12_mim); (**b**) Octamolybdate (Mo_8_) isomers and heptamolybdate (Mo_7_) in ball and stick (green: Mo, red: O) and polyhedral representations.

We report here synthesis and structure of a polyoxomolybdate-ionic liquid hybrid crystal. Deprotonatable 1-dodecylimidazolium ([C_3_H_4_N_2_(C_12_H_25_)]^+^ (C_12_im), Figure 1a) cations were employed to obtain C_12_im-polyoxomolybdate (C_12_im-Mo) hybrids (referred to as **1**). Recrystallization of C_12_im-Mo hybrids resulted in the formation of single crystals comprising δ-type isomer of octamolybdate ([Mo_8_O_26_]^4−^ (Mo_8_), Figure 1b) anion, C_12_im-δ-Mo_8_ (referred to as **2**). The weak interactions between C_12_im cation and δ-Mo_8_ anion were investigated, and anhydrous conductivity was estimated.

## 2. Results and Discussion

### 2.1. Syntheses of C_12_im-Mo Hybrids

**Figure 2:**
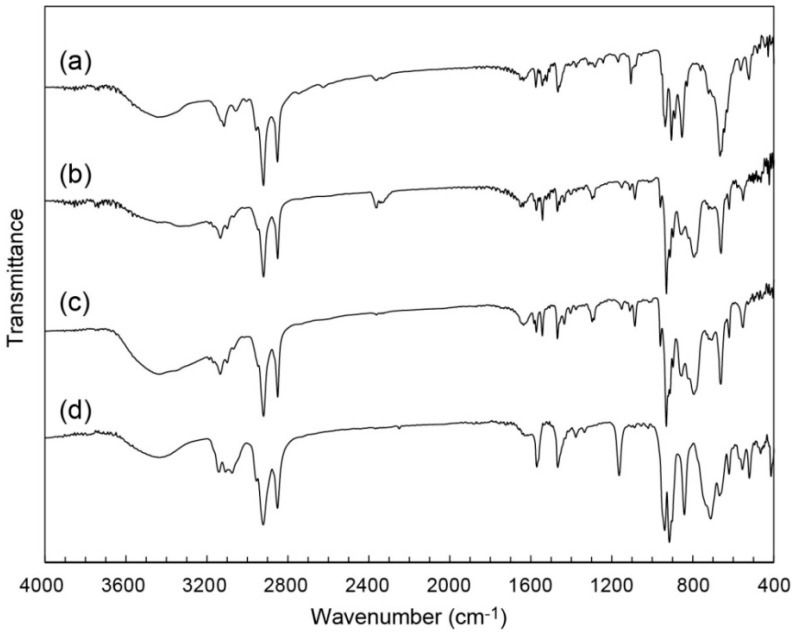
Infrared (IR) spectra of C_12_im-Mo hybrids: (**a**) C_12_im-Mo hybrid (**1**) obtained from C_12_im-N; (**b**) C_12_im-δ-Mo_8_ (**2**) obtained from C_12_im·Cl; (**c**) **2** after recrystallization; (**d**) C_12_mim-β-Mo_8_ (**3**) obtained by using C_12_mim.

**Figure 3:**
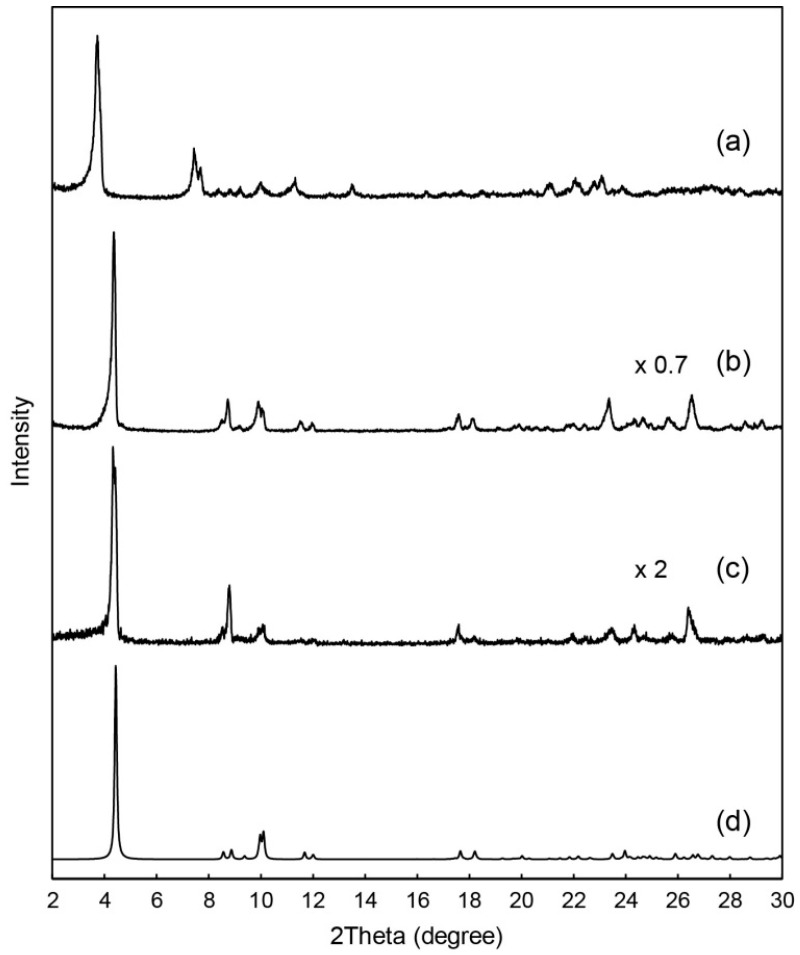
Powder X-ray diffraction (XRD) patterns of C_12_im-Mo hybrids: (**a**) **1** obtained from C_12_im-N; (**b**) **2** obtained from C_12_im·Cl; (**c**) **2** after recrystallization; (**d**) Calculated pattern of **2** using the structure obtained by single-crystal XRD.

As-prepared C_12_im-Mo hybrids were obtained as insoluble precipitates from aqueous solution of sodium molybdate (pH = 3.6) in 50%–65% yield (based on Mo). Figure 2 shows infrared (IR) spectra of as-prepared C_12_im-Mo hybrids. The structure of the C_12_im-Mo hybrids depended on the ionic-liquid species employed in the syntheses. Using neutral 1-dodecylimidazole (C_3_H_3_N_2_(C_12_H_25_), denoted as C_12_im-N) as surfactant source resulted in the formation of C_12_im-Mo hybrid of **1**. The IR spectrum of **1** (Figure 2a) showed characteristic peaks in the range of 400–1000 cm^−1^, indicating conceivable presence of heptamolybdate ([Mo_7_O_24_]^6−^, Mo_7_) in the hybrid [58,59]. C_12_im-N was acidified to form the C_12_im cation when added into the acidified sodium molybdate solution, and pH value will rise to cause the formation of the Mo_7_ anion [58,59]. On the other hand, utilizing the C_12_im cation prepared by prior neutralization of C_12_im-N with hydrochloric acid led to C_12_im-Mo hybrid of **2**, which contained α- or δ-type Mo_8_ anion (Figure 2b) [47,58,59]. The α- and δ-type Mo_8_ isomers are difficult to distinguish only by IR spectra, since they have similar molecular structures except for some elongated Mo-O bonds of the δ-Mo_8_ anion (represented in broken lines in Figure 1b). These C_12_im-Mo hybrids of **1** and **2** exhibited distinct powder X-ray diffraction (XRD) patterns (Figure 3a,b), indicating the formation of pure crystalline compounds having different structures.

Recrystallization of both **1** and **2** enabled us to obtain single crystals, which were identified to possess the same molecular and crystal structure as **2**, revealed by IR spectrum (Figure 2c) and powder XRD pattern (Figure 3c) of the single crystals. During the recrystallization process, the dissolved Mo_7_ anion from **1** will change to α- or δ-Mo_8_ in the solution [59,60], which reprecipitated into the single crystals of **2** (Figure 2a,c and Figure 3a,c). On the other hand, the structure of **2** was retained before and after the recrystallization process (Figure 2b,c and Figure 3b,c). Interestingly, the presence of AlCl_3_·6H_2_O under the recrystallization process was necessary to obtain suitable single crystals, as in the case when 1-dodecyl-3-methylimidazolium cation ([(C_12_H_25_)C_3_H_3_N_2_(CH_3_)]^+^, C_12_mim) and β-type Mo_8_ anion were hybridized to form C_12_mim-β-Mo_8_ (referred to as **3**) [45]. No presence of AlCl_3_·6H_2_O resulted in the formation of precipitates or crystals with worse quality. This implies that the hydrated Al^3+^ ion allows slow crystallization. In addition, the crystallization of **2** from **1** also requires the presence of AlCl_3_·6H_2_O, which may promote the structural conversion from Mo_7_ to α- or δ-Mo_8_.

The powder XRD patterns of as-prepared and recrystallized **2** measured at ambient temperature (Figure 3b,c) were almost the same in the peak position as the pattern calculated from the results of single crystal X-ray analysis (Figure 3d). This indicates that **2** was obtained as a single phase, being consistent with the results of elemental analyses. Slight differences in the peak intensity and position of the patterns may be due to the difference in the measurement temperature (powder: ambient temperature, single crystal: 93 K), and to preferred orientation derived from the predominant layered structure of **2**.

### 2.2. Crystal Structure of C_12_im-δ-Mo_8_ (**2**)

The X-ray structure and elemental analyses revealed the formula of **2** to be [C_3_H_4_N_2_(C_12_H_25_)]_4_[δ-Mo_8_O_26_] (Table 1). The crystal structure contained δ-type Mo_8_ anion with no solvent of crystallization, which was consistent with the IR spectrum (Figure 2c). Four C_12_im cations (1+ charge) were associated with one δ-Mo_8_ anion (4-charge) due to the charge compensation. **2** contained only the C_12_im cation as counter cation, being similar to the hybrid crystal of **3** [45].

The IR spectra of **2** exhibited the characteristic peaks of δ-Mo_8_ (Figure 2b,c), which contrasted with that of **3**, which consists of the β-Mo_8_ anion (Figure 2d). This difference in the Mo_8_ isomer structures is notable, since C_12_mim cation preferred β- or γ-type Mo_8_ anion [45,46]. The difference in the Mo_8_ isomers seems to depend on the difference in the hydrophilic moiety of ionic-liquid surfactants. C_12_im has no methyl group in the imidazole ring, while C_12_mim has one methyl group. The charged imidazolium moiety of C_12_im or C_12_mim strongly interacts with Mo_8_ anions. The difference in the bulkiness of the hydrophilic moiety and in the ability to form a strong N–H···O hydrogen bond (see below) may result in the formation of different Mo_8_ isomer structures in **2** and **3**.

**Figure 4:**
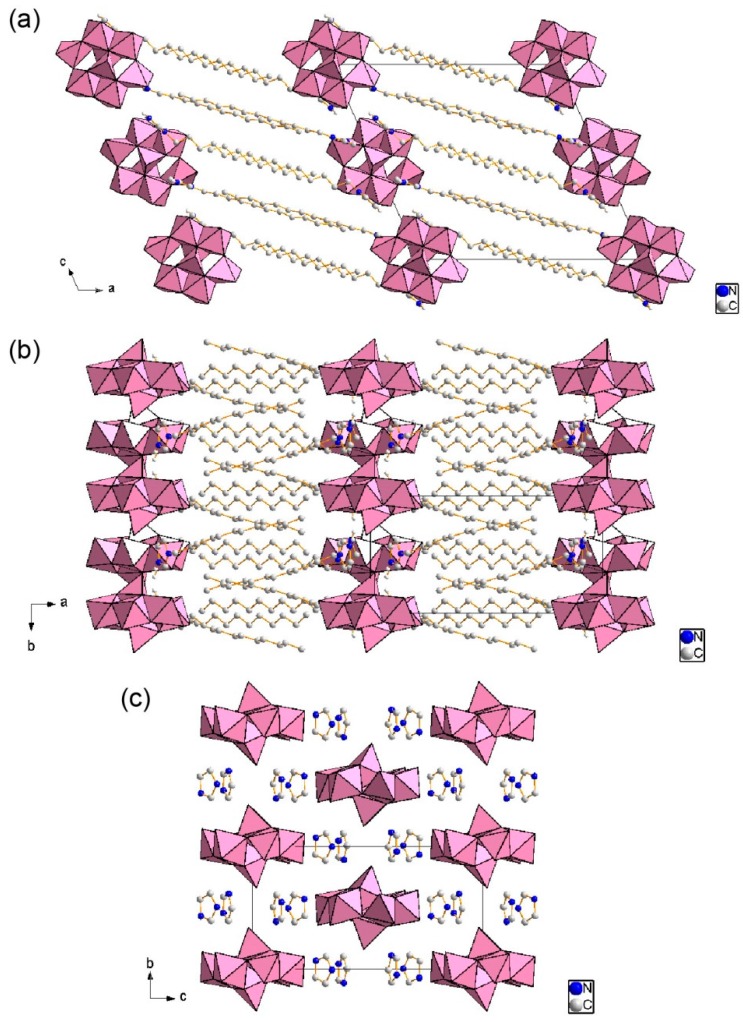
Crystal structure of **2** (C: gray, N: blue; δ-Mo_8_ anions in polyhedral representations). H atoms are omitted for clarity; (**a**) Packing diagram along *b* axis; (**b**) Packing diagram along *c* axis; (**c**) Molecular arrangements in the inorganic layers. The dodecyl groups are omitted for clarity.

**Figure 5:**
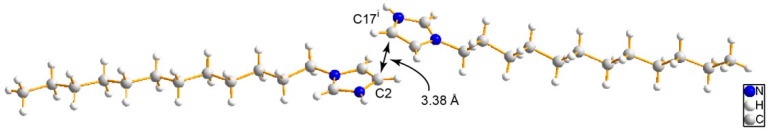
View of crystallographically independent C_12_im cations. Symmetry code: (*i*) −*x*, −0.5 + *y*, 0.5 − *z*.

Figure 4 shows the crystal structure of **2**. The crystal packing consisted of alternating δ-Mo_8_ inorganic monolayers and C_12_im organic bilayers with an interlayer distance of 19.9 Å (Figure 4a,b). The hydrophilic heads of C_12_im penetrated into the δ-Mo_8_ layers to isolate each δ-Mo_8_ anion (Figure 4c), being similar to that in the crystal of **3** [45]. The two crystallographically independent C_12_im cations formed a paired structure (Figure 5). They had a slight overlap of the imidazole rings, indicating the presence of a π–π stacking interaction (distance of C2–C17 bond between the imidazole rings: 3.38 Å). All C–C bonds of the C_12_im in **2** had *anti* conformation. These conformations of the imidazole rings and long alkyl chain were similar to the crystal of **3** [45].

In the crystal structure of **2**, two types of hydrogen bond were observed, namely N–H···O and C–H···O hydrogen bonds [61]. Most hydrogen bonds were formed at the interface between the δ-Mo_8_ and C_12_im layers. The N–H···O hydrogen bonds were derived from protonated nitrogen atom of imidazole ring in the C_12_im cation. The N···O distances of the N–H···O hydrogen bond in **2** were 2.89–3.09 Å (mean value: 2.97 Å), indicating the presence of strong hydrogen bonds. The C–H···O hydrogen bond in **2** exhibited C···O distances of 2.87–3.86 Å (mean value: 3.42 Å), which was similar to the C···O distances in **3** (3.04–3.85 Å, mean value: 3.42 Å) [45,62].

### 2.3. Conductivity of C_12_im-δ-Mo_8_ (**2**)

**Figure 6:**
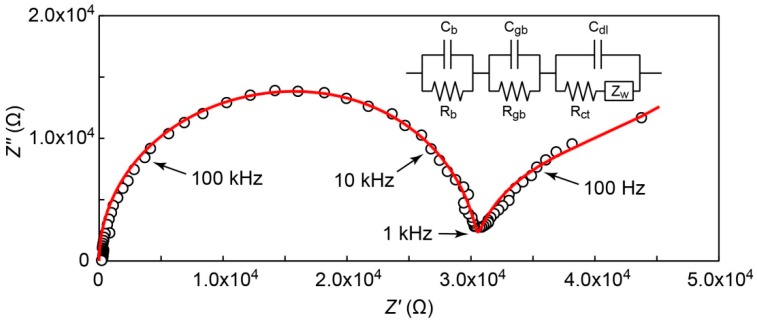
Nyquist spectrum (open circles) of as-prepared C_12_im-δ-Mo_8_ (**2**) at 443 K and simulated spectrum (red line) based on an equivalent electronic circuit in the figure. The parameters obtained by the fitting (see text) are as follows: *R*_b_ = 1.85 × 10^4^ Ω, *R*_gb_ = 1.2 × 10^4^ Ω, *R*_ct_ = 1.1 × 10^4^ Ω, *C*_b_ = 1.0 × 10^−8^ F, *C*_gb_ = 6.0 × 10^−9^ F, *C*_dl_ = 3.0 × 10^−6^ F, σ = 2.2 × 10^4^ Ω·s^−1/2^ (*Z*_w_ = $\left(1-j\right)\sigma /\sqrt{\omega }$).

Figure 6 shows an impedance spectrum for as-prepared **2** at 443 K under anhydrous atmosphere. As mentioned above, **2** retained both molecular and crystal structures before and after the recrystallization process. The spectrum showed a suppressed half circle in the high- and medium-frequency regions and an inclined line in the low-frequency region. The suppressed half circle will be derived from two overlapped semicircles due to bulk and grain boundary elements [48,49]. The linear part in the low-frequency region would result from combination of charge transfer resistance and Warburg impedance related to the diffusion of the carrier. The equivalent circuit employed here is shown in Figure 6 (inset). It consists of bulk resistance and capacitance (*R*_b_ and *C*_b_), grain boundary resistance and capacitance (*R*_gb_ and *C*_gb_), and charge transfer resistance (*R*_ct_) along with double layer capacitance (*C*_dl_). *Z*_W_ represents the Warburg impedance. The red line in Figure 6 represents simulated data with the equivalent circuit, which successfully reproduced the measured impedance spectrum. The estimated value of *R*_b_ was 1.85 × 10^4^ Ω, from which the conductivity of **2** was calculated to be 5.5 × 10^−6^ S·cm^−1^. This anhydrous conductivity is due to the residual proton in the bulk solid of **2** derived from the deprotonatable C_12_im cation, since **2** contained no molecule of crystallization nor small counter cation as a plausible source of carrier. The proton attached to the imidazole ring in C_12_im will be dissociated at the intermediate temperature of 443 K. Although the value of the anhydrous conductivity is not high enough, conductive polyoxometalate-surfactant hybrid crystals would pave a way to another class of anhydrous proton conductors at intermediate temperatures.

## 3. Materials and Methods

### 3.1. Materials and Genaral Methods

All chemical reagents except for imidazolium surfactant were obtained from commercial sources (Wako, Osaka, Japan and TCI, Tokyo, Japan, the highest grade). 1-dodecylimidazole (C_12_im-N) and its hydrochloric-acid salt ([C_3_H_4_N_2_(C_12_H_25_)]Cl, C_12_im·Cl) were prepared according to the literature [63].

IR spectra (as KBr pellet) were recorded on a Jasco FT/IR-4200ST spectrometer (Tokyo, Japan). Powder X-ray diffraction (XRD) patterns were measured with a Rigaku MiniFlex300 diffractometer by using Cu Kα radiation (*λ* = 1.54056 Å) at ambient temperature.

Conductivity measurements were carried out by alternating current (AC) impedance method in a frequency range from 20 to 1.0 × 10^7^ Hz using a Wayne Kerr 6510P inductance-capacitance-resistance (LCR) meter. Pelletized powder samples (10 mm in diameter, 0.79 mm in thickness) were sandwiched with Pt electrodes, and the impedance was measured under a dry N_2_ atmosphere at 443 K.

### 3.2. Synthesis

As-prepared C_12_im-Mo hybrid of **1** was precipitated by adding ethanol solution of C_12_im-N (0.2 M, 10 mL) to Na_2_MoO_4_·2H_2_O aqueous solution (0.4 M, 10 mL), which was adjusted to pH 3.6 with 6 M HCl. The precipitates were isolated by filtration, and dried in the ambient atmosphere to obtain colorless powder of **1** in a yield of 64%. Melting point: 463 K. IR (KBr disk): 936 (m), 905 (s), 889 (m), 853 (s), 829 (w), 761 (w), 722 (w), 665 (vs), 644 (m), 563 (w), 523 (w), 481 (w), 444 (w), 428 (w) cm^−1^. 

C_12_im-δ-Mo_8_ hybrid of **2** was prepared as follows: to aqueous solution of Na_2_MoO_4_·2H_2_O (0.4 M, 10 mL) acidified to pH 3.6 with 6 M HCl was added ethanol solution of C_12_im-N (0.2 M, 10 mL) neutralized by 1 M HCl (1.7 mL). The resulting precipitates were isolated by filtration, and dried in the ambient atmosphere to obtain colorless powder of **2** in a yield of 59%. Using ethanol solution of C_12_im·Cl (0.2 M, 10 mL) instead of the acidified C_12_im-N solution gave the same hybrids (yield: 52%). Anal.: Calcd for C_60_H_116_N_8_Mo_8_O_26_: C: 33.78, H: 5.48, N: 5.25%. Found: C: 33.14, H: 5.21, N: 5.11%. Melting point: 501 K. IR (KBr disk): 960 (w), 930 (s), 912 (s), 898 (m), 856 (m), 795 (s), 721 (w), 661 (s), 620 (w), 552 (w), 499 (w), 464 (w), 422 (w) cm^−1^.

Colorless platelet crystals of **2** were obtained as follows: acetonitrile solution (15 mL) of the as-prepared C_12_im-Mo hybrid (**1** or **2**, 0.03 g) and AlCl_3_·6H_2_O (0.02 g) was kept at 323 K for one day. The resulting supernatant was kept at 303 K for a few days, and then evaporated at room temperature to obtain colorless plates of **2** in ca. 30% yield. Anal.: Calcd for C_60_H_116_N_8_Mo_8_O_26_: C: 33.78, H: 5.48, N: 5.25%. Found: C: 34.49, H: 5.66, N: 5.30%. Melting point: 493 K. IR (KBr disk):960 (w), 930 (s), 912 (s), 898 (m), 855 (m), 795 (s), 707 (w), 661 (s), 620 (w), 553 (w), 498 (w), 472 (w), 457 (w) cm^−1^.

### 3.3. X-ray Crystallography

Single crystal XRD measurements were performed on a Rigaku Saturn70 diffractometer (Tokyo, Japan) using graphite monochromated Mo-Kα radiation (*λ* = 0.71075 Å). Diffraction data were collected and processed with CrystalClear [64]. The structure was solved by direct methods [65]. The refinement procedure was performed by the full-matrix least-squares using SHELXL Version 2014/7 [66]. All calculations were performed using the CrystalStructure [67] software package. All non-hydrogen atoms were refined anisotropically, and the hydrogen atoms on C atoms were refined using the riding model. Further details of the crystal structure investigation may be obtained free of charge from the Cambridge Crystallographic Data Centre, 12 Union Road, Cambridge CB2 1EZ, UK; fax: (+44)-1223-336-033; or E-Mail: deposit@ccdc.cam.ac.uk (CCDC 1472277).

## 4. Conclusions

Polyoxomolybdate hybrid crystals were successfully obtained by employing deprotonatable ionic-liquid cation, 1-dodecylimidazolium ([C_3_H_4_N_2_(C_12_H_25_)]^+^, C_12_im). The crystal contained δ-type octamolybdate ([Mo_8_O_26_]^4−^, Mo_8_), being different from the case of crystals comprising methylimidazolium surfactant having no dissociative proton. The crystal structure of C_12_im-δ-Mo_8_ consisted of alternate stacking of the δ-Mo_8_ layers and C_12_im layers. The hydrophilic moiety of the C_12_im cation formed N–H···O and C–H···O hydrogen bonds between the Mo_8_ anions, and the presence of the N–H···O hydrogen bonds suggests the formation δ-type Mo_8_ in the C_12_im-δ-Mo_8_ crystal. The C_12_im-δ-Mo_8_ crystal exhibited anhydrous conductivity of 5.5 × 10^−6^ S·cm^−1^ at 443 K presumably due to the proton dissociated from the protonated C_12_im cation, which is promising for the exploration of anhydrous proton conductors working at an intermediate temperature region.

## Figures and Tables

**Table 1:** Crystallographic data.

Compound	C_12_im-δ-Mo_8_ (2)
Chemical formula	C_60_H_116_N_8_Mo_8_O_26_
Formula weight	2133.13
Crystal system	monoclinic
Space group	*P*2_1_/*c* (No. 14)
*a* (Å)	21.859(4)
*b* (Å)	10.0395(18)
*c* (Å)	20.683(4)
α (°)	90.0000
β (°)	114.307(2)
γ (°)	90.0000
*V* (Å^3^)	4136.7(14)
*Z*	2
ρ_calcd_ (g·cm^−3^)	1.712
*T* (K)	93
*μ* (Mo·Kα) (mm^−1^)	1.244
No. of reflections measured	27938
No. of independent reflections	7550
*R*_int_	0.0463
No. of parameters	460
*R*_1_ (*I* > 2*σ*(*I*))	0.0422
*wR*_2_ (all data)	0.1292

## Supplementary Materials

Supplementary materials can be found at http://www.mdpi.com/1422-0067/17/7/994/s1, cif file of C_12_im-δ-Mo_8_ (**2**).
